# Research on optimization of real-time efficient storage algorithm in data information serialization

**DOI:** 10.1371/journal.pone.0260697

**Published:** 2021-12-16

**Authors:** Bin Huang, You Tang

**Affiliations:** 1 Electrical and Information Engineering College, JiLin Agricultural Science and Technology University, Jilin, China; 2 Smart Agricultural Engineering Research Center of Jilin Province, Jilin, China; Hankuk University of Foreign Studies, REPUBLIC OF KOREA

## Abstract

**Background:**

Along with the vigorous development of Internet technology, increasing the functions of the various types of equipment, network communication easy and diversity, at the same time, the amount of data is very huge, under the network bandwidth limitations, through long lead to a data need to be cut into more, one by one, transfer times, information delay problems.

**Results:**

Aiming at the problems of poor data integrity, low efficiency and poor serialization efficiency of traditional data storage information, this article introduces Protobuf technology to study the serialization of data storage information. The serpentine gap method is used to complete the allocation interval of the sequence nodes, so that the working state and the resting state always maintain a dynamic balance. According to the first-level rules, the storage data of the completed target node is obtained, and the grammatical structure and the semantics of the target data are analyzed, Meanwhile corresponding correspondences are established, and the data storage information is serialized. In order to verify the effectiveness of Protobuf’s data storage information serialization method, a comparative experiment is designed. By using three methods of HDVM, Redis and Protobuf to serialize JSON data, the comparative analysis shows that HDVM has the longest processing time and Protobuf has the shortest processing time, and the data integrity is not affected. The simulation data shows that the Protobuf serialization method has short conversion time, high space utilization, and the Obvious advantages in correctness and integrity. It is vary suitable for serialization of JSON data in the case of limited bandwidth.

## Introduction

In wireless sensor monitoring network with low requirement on real-time monitoring data, in order to save energy, nodes temporarily store monitoring data and transmit data when necessary in order to save energy. Such nodes are called storage nodes [1,2]. The selection of storage nodes directly affects the efficiency of query data and the energy consumption of nodes in the process of query and data transmission.

Most B/S architecture software today uses JSON [3,4] as a format for information transfer, and the simplicity and clarity of the hierarchy make JSON an ideal data exchange language. JSON is short for JavaScript Object Notation. JSON is a syntax for storing and exchanging data, and it uses JavaScript object notation to write text. Because when data is exchanged between the browser and the server, it can only be text, and JSON is text, it can be easily transferred between server browsers and used as the data format for any programming language. So we can convert any JavaScript object to JSON and send the JSON to the server. JSON is easy for people to read and write, and easy for machines to parse and generate, However, under the limitation of network bandwidth, the long length of data leads to the fact that a piece of data needs to be cut into several pieces and transmitted many times one by one, which causes the problem of information delay. For this, it is needed that a way to serialize JSON data efficiently can compress the information to a length suitable for a single transfer, and serialize or deserialize extremely quickly. Due to its features of being good, efficient, and easy to develop, it is Protobuf that is ideal for serializing JSON data in bandwidth-constrained situations.

With the vigorous development of Internet technology, the functions of various types of various types of equipment are increasing day by day, and the network communication modes become convenient and diversified, which cause that the amount of data generated is extremely huge.

Therefore, how to ensure the normal storage of gateway data has become one of the core tasks of scholars in related fields [5,6].Traditional serialization techniques, such as XML [7] and JSON [8,9], have been widely used in various network information storage and exchange centers due to their good readability. However, both XML and JSON need to change the format and redeploy the program.

Relevant scholars put forward the following solutions: Canhua Peng REDIS [10] regarded REDIS as valuable data storage. By optimizing the REDIS cache system algorithm, it can improve efficiency and hardware capacity of the cache, It also imports the guide installation in modern statistics theory and constructs pseudo number based on gauss random phase. Then it configures serialization algorithm, and finally the stack method is used to import the stored data into disk. At the same time, Huffman code can store the communication cache data. However, in practical application, this method has high readout rate and poor accuracy, so it is difficult to store information completely. Haidong Fu [11] proposed an HDVM serialization structure based on data set index, which selects triple connection matrix from data set, and uses the structure of correlation vector, predicate vector and object matrix to serialize hardware. It can reduce the number of iterations of correlation data, and improve the speed of information serialization. However, the algorithm requires high performance of the system and is difficult to be widely used in real life. Literature [12] proposes a data-centered storage method, which stores the data to the corresponding nodes through some mapping method according to the attribute value of the data, so that each node can only store one type of data, and the data can be obtained from the corresponding nodes through the corresponding mapping method when querying. For example, Combs [13], Double Ruling [14], SCOOP [15] and GHT [16] algorithms affirm that all nodes of the network have the same status and store data and index information on all nodes in a balanced way. In the GHT algorithm, there is only one storage node for each type of event, so communication bottleneck and hot spot phenomenon will occur. The hash position obtained by the hash function may not have a node; Also, the energy overhead of data storage and querying is not taken into account.

Therefore, the Protobuf serialization method is introduced to study the serialization integrity and efficiency evaluation of data storage information. The serialization data structure is more concise and the method has obvious advantages in performance. In the data storage procedure, the content is only the data itself and label, without irrelevant names and boundary characters. Under the same conditions of network data, Protobuf can save the system space (binary mode) to the greatest extent, and use a fast and flexible way to store information. Users can even build a specific sequence structure, use the transformed code to read its structural information, and re-establish the structure without changing the deployment program to achieve reliable data storage and information serialization.

## ROTOBUF principle

**Fig 1:**
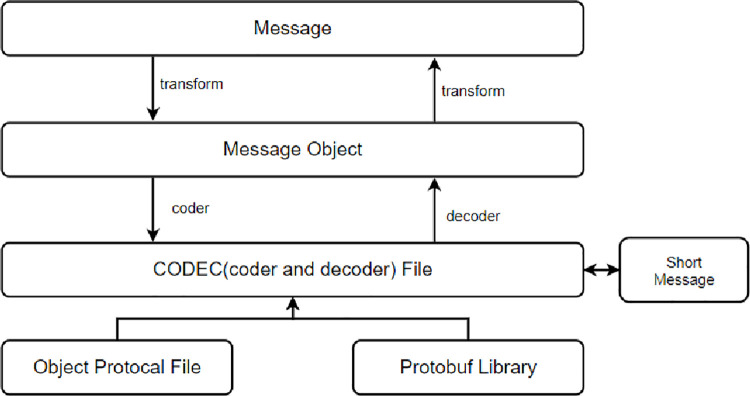
Code flow.

The full name of Protobuf [17–19] is Protocol Buffer, which is Google’s serialization model for open source goals on the web. Regardless of language and information platform limitations, it can be used for various types of communication protocols as a data series tool for information storage due to its excellent scalability and communication. It provides a good environment for data communication and exchange between networks. Codec flow is shown in Fig 1.

**Fig 2:**
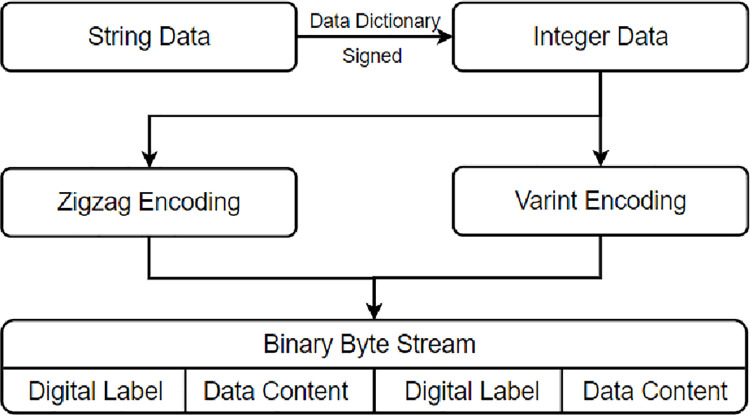
Data processing flow.

The encoding of the data content is selected according to the data type, and the selection method is shown in the Fig 2 below.

Protobuf can be understood as the serialization structure that needs to be built to store any proto file in which the message information is part of the logic of the data. Then, through its own compiler, the Protobuf file is converted to a fixed computer language, including Java, C++, Python and other categories. In this way, any field can be read easily and quickly, and it can be serialized or deserialized with access to complete the acquisition of message information.

PROTOBUF data structures are different from XML and JSON in that JSON data structures are defined as name/value pairs when they are defined and transmitted, including names, values, and boundary characters. It has one or more specific fields, each of which has a modifier, value type, value name, and field label. The data representation is very compact, so it can see that Protobuf uses less storage to transmit data and the sales volume is relatively high.

**Fig 3:**
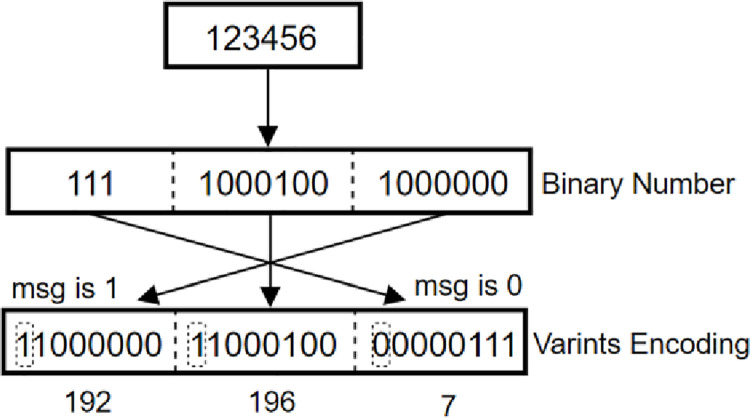
Varints coding procedure analysis.

The encoding that Protobuf uses is the compact number Varints [20], Users can bind the initial data to the associated numbers and store them in the same location, If it is an integer and the number is of type INT32, Varints can be represented with only one byte. The highest bit of each byte in Varints indicates whether this byte is the last byte. 1 means that the subsequent byte also means the number, and 0 means that this byte is the last byte. For example, 123456 uses the Varints encoding method of 11000000 11000100 00000111Details are shown in Fig 3 below.

In the image, t if the highest MSG of the first byte is 1, it indicates that part of valid data still exists in the following byte; if MSG is 0, it indicates that the last few digits in the following byte are the last significant digits, and then the MSG of the highest bit can be removed. After Variants encoding, the data takes up less system space, and the digits no more than 127 can be represented by a single byte. A number larger than 0xfffffff would need to be represented in five bytes, but such a large number would not exist under normal operations.

Protobuf serialization can minimize the byte space occupied by information and reduce the requirement of system space performance under the premise of ensuring the integrity of data information.

**Fig 4:**
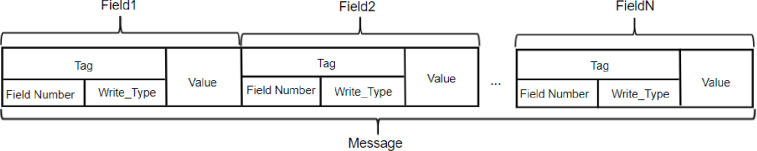
Tag uses Varints encoding.

All data is serialized in binary form, with any byte closely linked by semantic syntax. The Tag USES Varints encoding are shown in Fig 4

In Fig 4, a message contains multiple fields. When serializing the message, the byte length required for serializing all the fields is calculated first. In ProtoBuf, the number of bytes taken by each type of field is known (except Bytes and String), and you just need to sum it up. Each field outputs byte data of int32(tag, type) and value. Each field has a unique number tag that represents its index position and write_type is the type of the field. The message is serialized into a binary data stream consisting of a series of key-value pairs.

One of the core technologies of Protobuf is serialization and deserialization. Serialization refers to the process of converting a data structure or object into a binary string, while deserialization is the reverse operation of the above process, converting the binary string generated during the serialization process into a data structure or object. The serialization process does not need delimiters to separate fields, and each field is stored very compact, so storage space utilization is very high; If a field is not set with a field value, then the field is not present in the serialization data at all, that is, it does not need encoding. The Protobuf deserialization process is as follows: (1) Call parse From(input) of the message class to resolve the binary byte data stream read from the input stream;(2) Read the parsed data into the corresponding structure types of Java, C++ and Python according to the specified format. Since deserialization is the inverse process of serialization, there is no need for complex lexical syntax analysis, and the parsing process can be completed only by simple decoding [21,22].

## Data storage algorithm analysis

The snake gap method is most commonly used in computer networks, which divides different areas by network, screens out nodes with large storage space and close distance. The corresponding information of data is stored in it first, so it can reduc storage energy consumption and improve the effect.

### Network node allocation interval

The essence of the snake gap method [23,24] is the allocation interval of all nodes in the grid. Through time gap analysis, only two nodes in the global grid are in operation, and the rest nodes are at rest.

Firstly, the number of all nodes in the global grid and the distance between any node and the network center are calculated. Nodes are numbered in order of distance, with those closest to the center in the first place and those farther away in the last. The matrix T with m rows and n columns is set as the allocation interval for all nodes in the grid. In order to maintain the dynamic balance between rest and work, it is necessary to minimize the difference between m and n [25,26]. Then, the relationship between T and m, n and the number of all nodes N in the grid is shown in Formula (1).


$$\left\{ \begin{array}{c} m+n=N\\ m=[N/2] \end{array}\right.$$ (1)

Where, if N is even, then m = n = N/2; If N is odd, then m = N /2 and n = N-m.

The interval allocation of node working state T_ij_ is shown in Formula (2)

$$T_{ij}=\left\{ \begin{array}{l} i*n-n+j\;(i\;wsa\;odd)\\ i*n+i-j\;(i\;was\;even) \end{array}\right.$$ (2)

**Fig 5:**
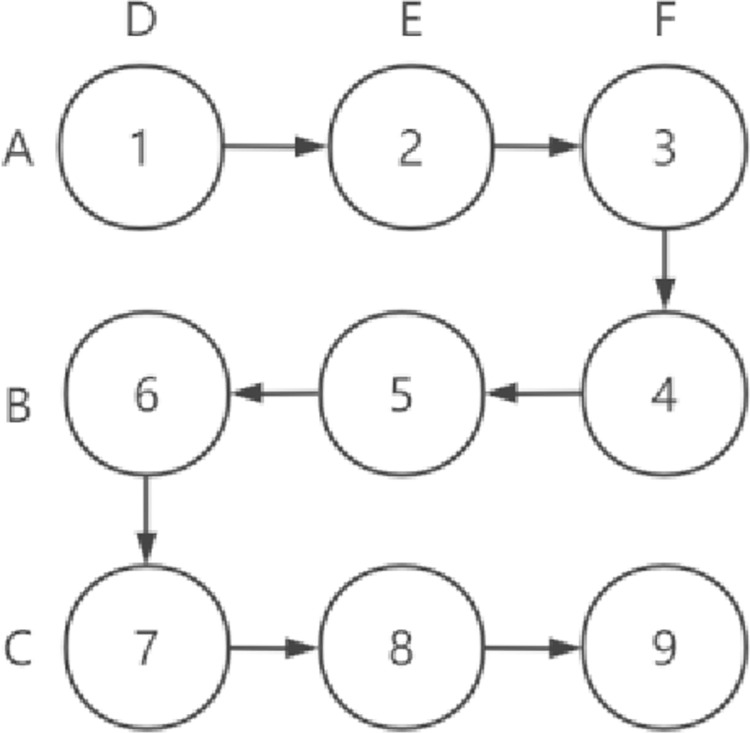
Schematic diagram of node working slot allocation in 3x3 matrix.

Suppose there are 6 nodes in the grid (A,B,C,D,E,F), n = 6, It can be obtained from Formula 1, m = 3,n = 3, The construction matrix is shown in Fig 5.

Time slots are allocated from left to right starting at Row 1, Column 1, and vertically to the next row if a matrix boundary is encountered, and time slots are allocated in the opposite direction. As shown in Fig 5, the time slot of Row1 from left to right is 1–3, and Row2 from right to left is 4–6, and so on. Each node is mapped to Row i or Column j, ensuring that two nodes are active in each slot. In matrix T, the elements in the corresponding row or column of the node represent the working time slot of the node. The active time slot of node A is 1–3, and the rest time slots are in sleep state. The active time slot of node D is 1,6,7. Snaketiming allocation method is adopted to ensure that nodes are always in working state when switching slots. For example, nodes A and D in slot 1 are in the working state. When slot 1 switches to slot 2, node D enters the sleep state, and node E enters the working state from the sleep state. Node A is always in the working state during the switching process. This allocation method can avoid the packet loss phenomenon during slot switching, and ensure the reliability of the network operation. Formula (3)shows the node working slot allocation more intuitively, transforming the matrix T into a matrix S with m+n rows and M *n columns. In the matrix, 1 represents the working state and 0 represents the rest state.


$$s=(\begin{array}{cccccccccccccccccc} 1 & 1 & 1 & 0 & 0 & 0 & 0 & 0 & 0 & 1 & 1 & 1 & 0 & 0 & 0 & 0 & 0 & 0\\ 0 & 0 & 0 & 1 & 1 & 1 & 0 & 0 & 0 & 0 & 0 & 0 & 1 & 1 & 1 & 0 & 0 & 0\\ 0 & 0 & 0 & 0 & 0 & 0 & 1 & 1 & 1 & 0 & 0 & 0 & 0 & 0 & 0 & 1 & 1 & 1\\ 1 & 0 & 0 & 0 & 0 & 1 & 1 & 0 & 0 & 1 & 0 & 0 & 0 & 0 & 1 & 1 & 0 & 0\\ 0 & 1 & 0 & 0 & 1 & 0 & 0 & 1 & 0 & 0 & 1 & 0 & 0 & 1 & 0 & 0 & 1 & 0\\ 0 & 0 & 1 & 1 & 0 & 0 & 0 & 0 & 1 & 0 & 0 & 1 & 1 & 0 & 0 & 0 & 0 & 1 \end{array})$$ (3)

Convert Row i’ of matrix T to Row i of matrix S, and Column j’ of matrix T to Row (j + m) of matrix S; Converts the element value T_i’j’_ of matrix T to the columns of matrix S. After transformation, the rows of matrix S correspond to (m + n) nodes in the grid, and the columns correspond to (m × n) working time slots. The value of matrix element S_*ij*_ is only 1 or 0. *S*_*ij*_ = 1 means that node i is in active state at the jth time slot. S_*ij*_ = 0 means that node i is in dormant state at the jth time slot, as shown in Formula (4).


$$S_{ij}=\left\{ \begin{array}{c} 1\;(Working)T_{ij}=1,1\leq i\leq m,1\leq j^{,}\leq n,\\ T_{i^{,}}=j\;or\;m<i\leq m+n,\\ 1\leq i^{,}\leq m\\ 0\;(Sleeping)\;other \end{array}\right.$$ (4)

### Data storage target node filtering

**Fig 6:**
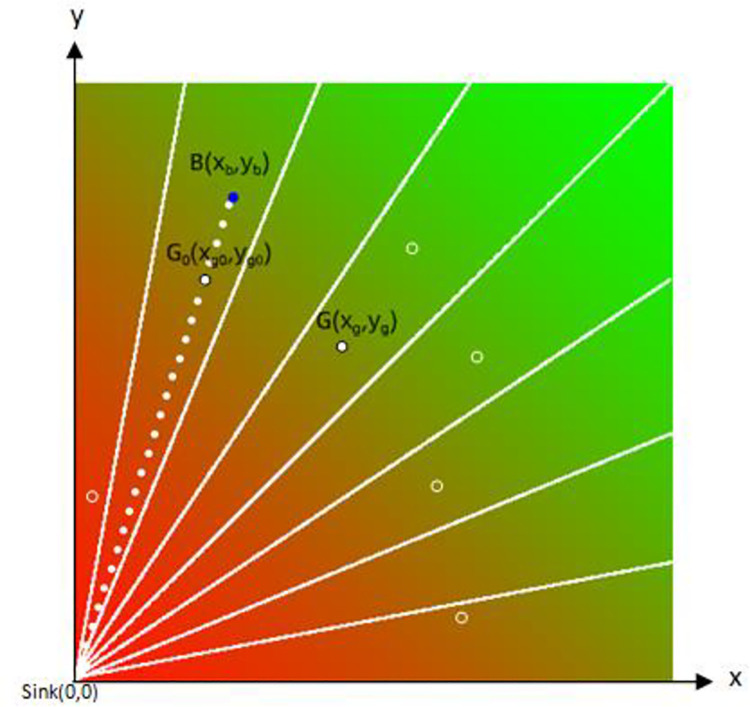
Data storage node distribution map.

The target node of data storage is filtered to achieve the initial data storage [27]. Fig 6 shows the distribution of data storage nodes.

In Fig 6, the observation node is B(Xb,Yb), and the corresponding position G(Xg,Yg) is found by using the hash method, and the homomorphic hash position G0 (Xg0,Yg0) is obtained from the observation points and the azimuth Angle of the hash, where G and G0 are within the same circular arc, and the observation point B and G0 are on the same extension line. The working node inG_0_ grid is regarded as the storage node of the initial data, Its coordinates are obtained from Formulas (5) and (6).


$$X_{g0}=\sqrt{\frac{(X_{g}^{2}+Y_{g}^{2})*X_{b}^{2}}{X_{b}^{2}+Y_{b}^{2}}}$$ (5)

$$Y_{g0}=\sqrt{\frac{(X_{g}^{2}+Y_{g}^{2})*Y_{b}^{2}}{X_{b}^{2}+Y_{b}^{2}}}$$ (6)

However, this method does not take into account the effect of network cache factor and cannot determine part of grid access mechanism. Therefore, the storage data life cycle is added to optimize the above algorithm. The initial data life cycle calculation is shown in Formula (7)

$$T=\alpha N_{s}+\beta N_{m}$$ (7)

Where, probability N_m_ is shown in Formula (8):

$$N_{m}=\frac{\sum_{i=1}^{n}P_{i}}{t}$$ (8)

In Formula (9):

$$P_{i}=\left\{ \begin{array}{c} 1,\;access\\ 0,\;not\;access \end{array}\right.$$ (9)

N _s_ represents the probability of accessing the original data again, and N_m_ represents the probability that the consumer determines the initial data cache entry, α and β are random numbers, which is convenient for calculation and has no practical significance. When the network storage node are scattered, their cache access probability is small, Therefore, in the process of life cycle calculation, only the import, processing and output regions are divided. So the formula for N S is shown in Formula (10):

Ns=Nsyφt+Nsmt+γNsct (10)

In Formula (10), T represents the time cycle ΔT consumed by importing initial data into system cache, N_SY_ represents the number of cached items that are accessed only, N_SM_ represents the number of initial data cache entries processed, N _SC_ is used to evaluate whether the data is new,φ (φ≥1) and γ (γ≥1) as parameters. Here, the value of N_sc_ is shown in Formula (11):

$$N_{sc}=\left\{ \begin{array}{c} 1,\;new\\ 0,\;read \end{array}\right.$$ (11)

The above formula can realize data storage and improve its storage efficiency.

### Data storage information integrity and efficiency evaluation method

Protobuf is a kind of information serialization, whose purpose is to convert target data into a form that can be stored or transmitted, and then read by code accessing or modifying the serial object. In other words, it is the process of converting user stored data sets into a Protobuf format that can reflect the semantic and logical framework of the target information. In this process, the data structure of the receiving target set is serialized, and the corresponding information is extracted from the initial data set to complete the serialization of data storage information. This is a data storage process, whose essence is to extract and transform the original data set in the network, then recompile the meaning of information according to some specific conditions, and encode the output according to its requirements. The main contents can be divided into three steps, namely, input, processing and output.

Step 1: Input. Using serialization to process the feature information in the data and infer the subsequent access patterns from the initial data structure Protobuf circulates the value in massage information after encoding Varints: Value& ~ 0 x7f = = 0 if the value of the value is less than 128, which is 1 byte can hold, is coming to an end at the end of message has to jump out of the loop, or the current value using a byte to hold the value & (0 x7f) | 0 x80 operation, the highest for 1 said there is behind the data, Continue the loop to determine the following data. So the top bit of the first byte of value is 1, which means there’s more data to follow, and if it’s 0, there’s no data, so it can reduce the running time and simplify the operation.

Step 2: Processing. In data processing, the information obtained from the initial data set will be recombined according to some specific conditions to clarify the meaning of semantic information, and the data will be output according to the format of the data set to be processed.

**Fig 7:**
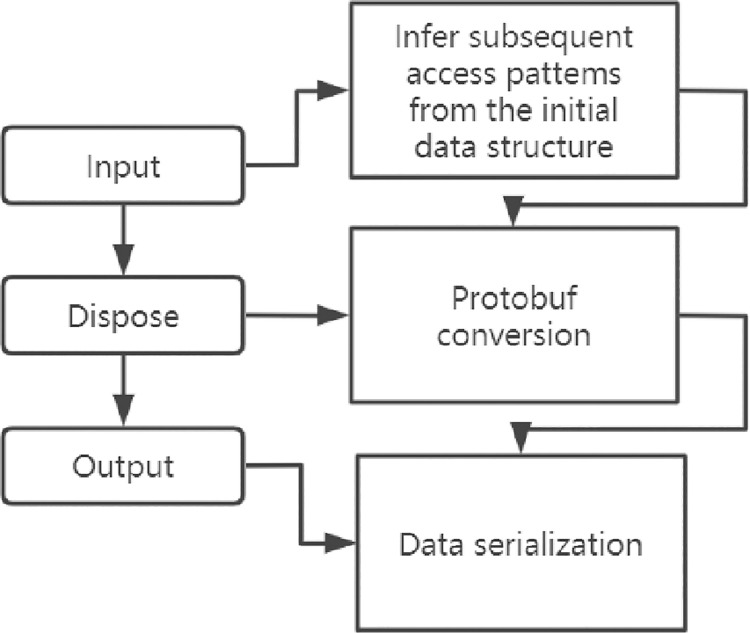
The Protobuf serialization step and its corresponding serialization form.

Step 3: Output. Serialize the output according to the target data set structure for the formal output. It is necessary to understand the target data structure and semantic description model. The above three steps can be analyzed in detail in Fig 7, and the serialization form corresponding to any step is shown in Fig 7.

### Initial data set structure

At present, network data is mostly static structure, so the precondition of the Protobuf serialization of data storage information is to comprehensively analyze its structure, However, the key to data structures is semantics and syntax.

#### (1) Semantic structure in the initial data

Semantics refers to value theory implied by data, including the meaning and expression of information. Before the data is serialized by Protobuf, the corresponding semantic information is obscure and uncertain, and its default value cannot identify or determine the object contained in the data through the network, so it cannot be understood and processed by the computer. When studying semantic structure, it is necessary to determine the corresponding relationship between different objects in the data (such as entity, number, attribute, etc.), and use the directed graph pattern to describe them. The purpose of studying semantic structure is to increase the matching degree between the initial data and the corresponding entity, establish the mapping relationship, and facilitate the computer extraction and recognition.

#### (2) Syntax structure in the initial data

As a specific condition of serialization, the syntactic structure can restrict the semantic structure and expression form in the process of encoding, transmission and exchange, so that the network can show the real world through data. Studying the syntax structure of the initial data can provide a more convenient way for the system to access.

### Structure of target data set

The Protobuf used in the target dataset is structured encoding, in other words, the semantic expression pattern will change according to the ontology or thesaurus used, so will the serialization file pattern. It is still based on the semantic and syntactic aspects.

#### (1) Research on semantic structure in target data

Analyze its semantic structure, understand the type, shape and attribute restriction of the target individual, and determine the vocabulary and hierarchical relationship contained in the semantics of serialization.

#### (2) Study on syntactic structure in target data

The representation of the target dataset can be found in the syntax, Although the important hierarchical relationships are presented in the form of directional diagrams, the parsing is presented in a tree hierarchy. When describing the meaning of a single attribute, Protobuf usually uses embedded components to reflect characters or to describe the logical and organizational relationship of framework.

### Build the mapping relationship between the initial data set and the target data set

The mapping relationship between the initial data set and the target data set based on semantic and syntactic structure needs to be constructed, so that the structure and template can be further provided for the Protobuf serialization transformation. Mapping relationships mainly exist at the semantic and syntactic levels of data.

Firstly, through the mapping relationship between the two semantic levels, the information attributes that must be preserved in data serialization are obtained.

Next, the initial data structure is analyzed, and the subsequent access patterns are determined, Then the expression is used to filter, and the structure is returned to the template.

Then, the data serialization transformation mode is determined through the mapping relationship between the two syntactic levels. According to the different semantic information contained in different data, the corresponding conditions and corresponding templates are set. The template rules correspond to the data serialization path, and the semantic explicit description of the transformation path is given by the Protobuf element. Also, the Protobuf serialization encoding is obtained.

Finally, based on the structural relations of syntax and semantics, the construction template of the object data set is defined. Templates are divided into multiple regions according to the semantic structure requirements of the target data set. Each template has different functions and can independently complete the corresponding import and output. Different templates complement and harmonize each other to complete the overall semantic and grammatical structure processing of the data.

### Protobuf serialization conversion

The design and format of Protobuf conversion data must correspond to the target content. The Protobuf overall file structure must be edited from top to bottom, and different template functions are carefully planned to guarantee that no bytes are lost during serialization. At the same time, in order to ensure that the byte attributes have no deviation, all attribute absolute values of the source node in the initial data are extracted and processed according to the constraint conditions, and the data Protobuf serialization transformation is implemented.

## Simulation data and results

In this experiment, Storm under the flow computing system was used to evaluate the performance of data storage information serialization integrity and efficiency evaluation method. Storm can effectively sort out all kinds of data streams, and conduct real-time cluster monitoring. It can distribute target data codes to task computers by Nimbus, and open or close task programs as required.

### Data storage image information serialization integrity verification

The image data samples were tested and compared after serialization program processing, it was found that the difference value of the two images was close to 0, and the integrity of transmission was good.

### Protobuf serialization time detection

**Fig 8:**
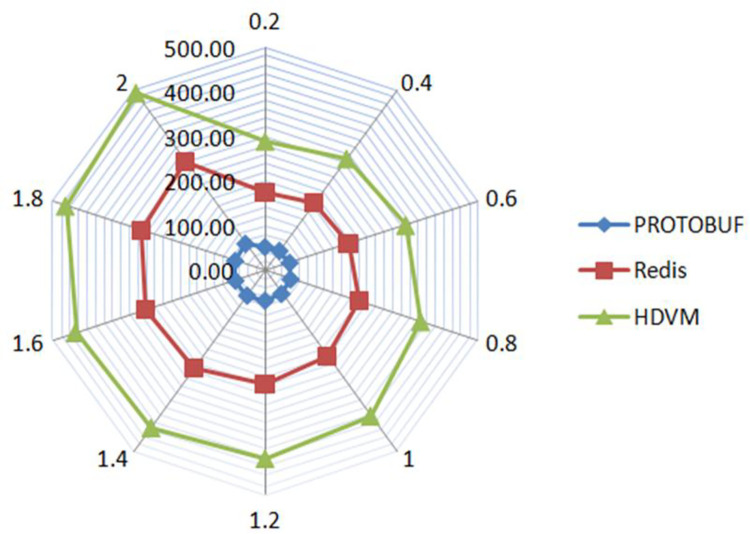
Data serialization processing time.

Take the user’s purchase information in a platform as the initial data set, and the bytes from left to right are user’s purchase ID, the product ID, and the purchase time. The user’s ID is recorded as the key value and the product ID as the value, and it is stored in the user’s corresponding ID file. Through the data set size, the serialization conversion time is detected as shown in Fig 8. The dataset of experimental results is shown in Table 1.

**Table 1:** Experimental data results.

Run time(s) Data size(GB)	Protobuf	Redis	HDVM
0.2	53.5975	175.5859	289.5255
0.4	55.5513	187.4627	309.5622
0.6	58.5847	196.5485	330.2945
0.8	60.5544	222.2155	365.5566
1	63.2541	238.1555	400.5448
1.2	66.4785	254.4548	419.5565
1.4	68.1554	269.4852	433.6554
1.6	69.9548	280.4822	446.5955
1.8	71.1654	291.1544	469.5545
2	74.9579	302.2458	491.5842

It can be seen from Fig 8 that the three methods have different processing times for data serialization. The serialization method based on relational matrix fluctuates within 290 ~ 590s and has the longest processing time. The processing time of the serialization method based on Redis varied between 180 ~ 280s, while that of the serialization method based on Protobuf varied between 60 ~ 80s, with the shortest time and the fastest processing speed.

## The conclusion

Through serialization and deserialization of files and images, it verifies that the serialization integrity of data storage information and the efficiency evaluation method have good accuracy and completeness, which solves the problem that the traditional technology cannot store and access the data in real time when processing unstructured data. In this paper, HDVM, Redis and Protobuf are used to carry out comparative experiments to serialize and deserialize files and images. The experimental results verify the serialization integrity and efficiency of data storage information. It is found that the HDVM method takes a longer time to process large information, while the Protobuf method takes a smaller time increment to process large information. Compared with Redis, Protobuf has a fast transformation speed, is not affected by the amount of serialized data, and the processing time increases linearly. The method has high efficiency and low requirements on system performance, which can meet the needs of most ordinary users. The evaluation method has good accuracy and integrity, which solves the problem that unstructured data cannot be stored and accessed in real time.
